# Factors associated with frailty in patients with neurodegenerative diseases

**DOI:** 10.1590/2317-1782/20212020214

**Published:** 2022-06-17

**Authors:** Rafaela Soares Rech, Marina Martins Pereira Padovani, Nathalia Flores Oliveira, Bruna Graciele Souza Alós, Annelise Ayres, Maira Rozenfeld Olchik

**Affiliations:** 1 Universidade Federal de Ciências da Saúde de Porto Alegre - Porto Alegre (RS), Brasil.; 2 Faculdade de Ciências Médicas Santa Casa de São Paulo - São Paulo (SP), Brasil.; 3 Universidade Federal do Rio Grande do Sul – UFRGS - Porto Alegre (RS), Brasil.; 4 Hospital de Clínicas de Porto Alegre - Porto Alegre (RS), Brasil

**Keywords:** Frailty, Aging, Swallowing Disorders, Cognition, Speech-Language and Hearing Science

## Abstract

**Purpose:**

To identify the factors associated with frailty in patients with neurodegenerative diseases.

**Methods:**

Cross-sectional study, whose sample consisted of 150 patients diagnosed with neurodegenerative diseases seen at a speech-language therapy clinic in a reference hospital in southern Brazil. A secondary exploratory analysis of the medical records of patients treated at this clinic between April 2016 and May 2019 was performed. The information collected was sex, age, education, type of neurodegenerative disease, time of disease, frailty (Edmonton Frail Scale-EFS), swallowing (Northwestern Dysphagia Patient CheckSheet-NDPCS, Eating Assessment Tool-EAT 10), and cognition (Mini-Mental State Examination-MMSE and Montreal Cognitive Assessment-MoCA). Continuous quantitative variables were analyzed using mean and standard deviation and categorical quantitative variables from absolute and relative frequency, as well as their association with the outcome using the Chi-square test. Crude and adjusted Prevalence Ratios were assessed using Poisson regression with robust variance. All statistical tests were considered significant at a level of 5%.

**Results:**

The significant factors associated with frailty were the presence of oropharyngeal dysphagia and altered cognitive performance. Individuals with frailty have a higher prevalence of oropharyngeal dysphagia (PR= 1.772(1.094-2.872)), while cognition alteration presented a lower prevalence (PR= 0.335(0.128-0.873).

**Conclusion:**

Oropharyngeal dysphagia can be an important clinical predictive factor for consideration in cases of frailty in patients with neurodegenerative diseases.

## INTRODUCTION

Neurodegenerative diseases, such as Alzheimer’s Disease, Parkinson’s Disease, Huntington’s Disease, Amyotrophic Lateral Sclerosis, Frontotemporal dementia, and Spinocerebellar Ataxias^(1)^, lead to reductions in the functionality of diverse bodily systems according to the specific characteristics of each pathology. These present similar characteristics: diminished mitochondrial function with increased oxidative damage, defects in the ubiquitin-proteasome system, abnormal presence of aggregated proteins, and alterations in the metabolism of iron^(2,3)^. These factors are part of a repetitive cycle, whereby the action of anyone can trigger damage to the neuron, which in its turn, “recruits” the other factors, to destroy the cell^(4)^.

Neurodegeneration is a debilitating and incurable condition, which appears in the literature with an exponential increase in prevalence^(1)^. The main physiological consequences related to this neural condition affect day-to-day activities and vital functions of the human being, mainly in tasks that demand motor, cognitive, swallowing, and speech complexity^(3,5)^. People of all ages can be diagnosed with neurodegenerative diseases, however, they are more prevalent in the elderly population^(6)^.

In this specific population, frailty is among the diverse aggravating factors consequent on neuronal damage and the impairment of human abilities^(7)^. Frailty has a multisystemic character and is associated with a phenotype that includes five main components: unintentional weight loss, self-reporting of fatigue and/or exhaustion, reduction in muscular force, slower gait speed, and low level of physical activity^(8,9)^. Currently, frailty is seen as a type of syndrome with a specific and independent base, being avoidable when identified early on, or postponed when there is intervention at the level of the causal conditions^(10,11)^.

Currently, studies seek to identify which aspects lead to more negative outcomes for neurodegenerative diseases. Some authors indicate that the incidence of frailty is greater in patients with Parkinson’s disease than in the general community and is associated with a more adverse outcome for the disease^(12)^. The same occurs with Alzheimer’s Disease and with the elderly independent of subcategories that would indicate specific at-risk populations, seeking to minimize frailty in the hope of modifying its impact on degenerative diseases and their consequences^(5,13)^.

Given the gaps in the indexed literature regarding the most prevalent characteristics of frailty and its associated factors in individuals with neurodegenerative diseases, as well as the consequences and particularities arising from the specific clinical situation for these populations, its predictors and clinical findings can optimize coordinated care and specific and interdisciplinary rehabilitation^(14)^. Additionally, it is believed that timely strategies can provide improvements to overall health and the quality of life for these individuals, as well as a reduction in healthcare costs^(15)^.

This study hypothesizes that oropharyngeal dysphagia is the main factor associated with frailty in patients with neurodegenerative diseases given the functional characteristics and existing aggravating factors in these cases in clinical practice. Therefore, we sought to identify the factors associated with frailty in patients with neurodegenerative disease.

## METHODS

This is a cross-sectional study with a consecutive intentional convenience sample with secondary data from medical records of patients with neurodegenerative diseases treated at a Speech-language therapy clinic at a reference hospital in the city of Porto Alegre – RS between April 2016 and May 2019. We excluded the data of patients who did not present a complete speech-language therapy evaluation or where the medical records did not present complete data.

In terms of estimating the necessary number of medical records to be analyzed, the calculation of the sample size was based on a study that associated cognition and frailty^(16)^. A 90% study power and a level of significance of 0.05 were adopted. To compensate for possible losses, the sample was increased by 10%. Therefore, the necessary sample size was defined at 150 individuals.

The contextual variables used in this study were: sex (female; male) age (20-47 years; 48-57 years; 58-65 years; 65 years or more), education (primary education; incomplete secondary education; secondary education; up to technical education; university education), cognition (normal; altered) and swallowing (normal; altered). The categories were established based on the sample distribution.

Frailty was determined based on the Edmonton Frail Scale^(17)^ (EFS), translated and validated for Brazilian Portuguese. Despite not being frequently used in neurodegenerative cases but rather in geriatric cases, this scale has been amply used worldwide and is considered a very broad, complete scale for its domains of investigation, with strong diagnostic accuracy, and easily applicable by diverse professionals^(18)^. Despite the absence of a scale for this specific population, this scale was used and the participants were diagnosed by speech-language therapists specialized in the area and who worked in the clinical sector of the hospital. It is a frailty evaluation scale for the elderly that includes nine domains: cognition, general state of health, functional independence, use of medications, nutrition, humor, urinary continence, and functional performance, investigated in 11 items. The score varies from 0 to 17 points. The scores for the analysis are 0-4 points, without any frailty; 5-6 points, apparently vulnerable; 7-8 points, light frailty; 9-10 points, moderate frailty; 11 or more, severe frailty.

The swallowing evaluation was made up of a clinical evaluation based on the *Northwestern Dysphagia Patient Check Sheet* (NDPCS) instrument, translated and adapted to Brazilian Portuguese^(19)^. It is a quick and functional clinical swallowing evaluation composed of 28 items divided into five categories: medical history, behavioral aspects, broad motor function, oral motor test, and observation during the swallowing tests. The swallowing tests were performed based on the free demand-supply of a liquid consistency, where signs suggestive of laryngotracheal penetration/aspiration according to the guidelines for the protocol used and the international recommendations for clinical speech-language therapy practice were adopted. The speech-language therapy diagnosis was considered normal when there were no risks, and altered when there was the possibility of penetration and/or laryngotracheal aspiration was observed. The categorization was performed according to sample distribution.

The self-perception evaluation for oropharyngeal dysphagia was performed based on the Eating Assessment Tool (EAT-10)^(20)^. It is a self-perception tool for the identification of the risk of dysphagia. Its aim is multidisciplinary identification and intervention as early as possible. It is made up of ten simple questions that provide information about functionality, emotional impact, and physical symptoms that dysphagia can generate in the individual. With a score of 3 or more, the participant was considered to be at risk of oropharyngeal dysphagia.

The cognitive evaluation was performed based on the Mini-Mental State Examination (MMSE)^(21)^ and Montreal Cognitive Assessment (MoCA)^(22)^, both validated for and translated to Brazilian Portuguese. It is a cognitive screening test that seeks to quickly verify cognitive function. The 11 tasks of the MMSE evaluate functions including spatiotemporal orientation, memory, attention, calculation, language, and constructive praxis. The 11 items are divided into two sections, one requiring verbal responses to questions regarding orientation, memory, and attention, and the other evaluating reading and writing, including abilities such as designation, verbal and written commands, and the capacity to copy a drawing. The score ranges from 0 to 30. Scores equal to or greater than 29 points for individuals with education greater than 11 years are considered median values within the range of normality; 28 points for individuals with 9 to 11 years of education; 26.5 points for individuals with 5 to 8 years of education; 25 points for 1 to 4 years of education and 20 points for illiterate individuals.

The MoCA evaluates eight functions: visuospatial apraxia, designation, memory, attention, language abstraction, and orientation. It has a total score of 30 points, with individuals who present a score equal to or above 24 points being considered cognitively normal. Both the MMSE and the MoCA were considered normal or altered according to the responses expected for each individual according to their education (if greater or lesser respectively).

### Statistical analysis

The descriptive analysis of the data was performed according to the distributions of each variable. For the analysis of the categorical variables, we used the absolute and relative frequencies for the analysis of the quantitative variables, applying the average and standard deviations due to the normality of the data, analyzed by the Kolmogorov-Smirnov test by the visual analysis of histograms for complementation. The Spearman correlation was applied to test the correlation of the variables and check for measurement bias. Given that all the variables presented a very weak (0 to 0.3), weak (0.3 to 0.5), and moderate (0.5 to 0.7) correlation value, they were added to the theoretical model for this study. Additionally, the presence of multicollinearity was evaluated using the estimates of the variance inflation factor (VIF), observing that the cutoff points are good (close to 1) indicating that the variables are not multicollinear. The Chi-square test was used to evaluate the associations in the variables studied with the level of significance of p ≤0.05. Calculating the relationships between the variables, and considering possible confusion factors, Poisson Regression with robust variance was used, based on the Prevalence Ratio (PR) and its respective confidence intervals of 95%. In the adjusted model, the theoretically relevant variables were included, with all the variables being included in the multivariate final model, given that this study had an exploratory character to identify how the variables behaved in neurodegenerative cases. The results were analyzed statistically using the Statistical Package for the Social Sciences (SPSS) version 18.0.

### Ethical Aspects

This study was approved by the Research Ethics Committee (REC) of the institution under process number 1803-23. All individuals involved in the research signed an Informed Consent Form.

## RESULTS

A total of 150 medical records were included in the study. Of these, 62 (41%) of the individuals had a genetic neurodegenerative disease, more precisely spinocerebellar ataxias followed by neuromuscular diseases 50 (33.7%), movement disorder diseases 21 (14%), and 17 (11.3%) cases of stroke.

Table 1 presents the characterization of the sample and its brute association (bivariate). The majority of the individuals were female 78 (52.0%). Additionally, the women presented a greater prevalence of frailty 41 (52.6%). The average age was 54.13 (± 8.41) years, and education, measured in years was from 8.74 (± 1.21) years and the average time of disease was 9.75 (± 2.34) years. The prevalence of alteration of swallowing diagnosed by the clinical evaluation was 42.0%, while cognitive alterations were present in 22.6% of the sample studied. The variables sex (p<0.000), alteration of swallowing (p<0.001), and cognitive alterations (p<0.001), remained associated with frailty.

**Table 1:** Sample characterization and scoring for frailty, swallowing, and cognition evaluations. Porto Alegre, 2018 (n=150)

**Variables**	**Without frailty**	**With frailty**	**p-valor**
**Sex**			<0.001
Female	37 (47.4%)	41 (52.6%)	
Male	52 (72.2%)	20 (27.8%)	
**Education**			0.227
Until primary education	25 (56.8%)	19 (43.2%)	
Until incomplete secondary education	18 (52.9%)	16 (47.1%)	
Until secondary education	24 (72.7%)	9 (27.3%)	
Until technical training, university and post-	21 (55.3%)	17 (44.7%)	
**Age**			0.243
20-47 years (1st quartile)	27 (67.5%)	13 (32.5%)	
48-57 years (2nd quartile)	17 (47.2%)	19 (52.8%)	
58-65 years (3rd quartile)	21 (60.0%)	14 (40.0%)	
65 years or more (4th quartile)	22 (56.4%)	17 (43.6%)	
**MMSE**			<0.001
Normal	51 (51.5%)	48 (48.5%)	
Altered	38 (74.5%)	13 (25.5%)	
**MOCA**			0.019
Normal	66 (56.9%)	50 (43.1%)	
Altered	23 (67.6%)	11 (32.4%)	
**Clinical evaluation of swallowing**			<0.001
Normal	60 (68.9%)	27 (31.1%)	
Altered	21(33.3%)	42 (66.7%)	

**Caption**: n – sample number; MMSE – Mini-Mental State Examination; MoCA – Montreal Cognitive Assessment.

Table 2 presents the ratios of the estimated multivariate prevalence based on the exploratory analysis for control of possible confusion factors. Oropharyngeal dysphagia and cognitive alteration remained significantly associated with frailty, with a prevalence of frailty being greater amongst those with dysphagia (RP=1.772(1.094-2.872)) and lower amongst those with cognitive alterations (RP=0.335(0.128-0.873)).

**Table 2:** Ratios of adjusted prevalence in neurodegenerative patients with frailty. Porto Alegre, 2018 (n=150)

**Variables**	**Frailty** **PR (CI)**	**p-valor**
**Sex**		
Female	1	-
Male	0.606 (0.339-1.083)	0.091
**Education**		
Until primary education	1	-
Until incomplete secondary education	1.158 (0.688-1.948)	0.581
Until secondary education	0.706 (0.307-1.624)	0.413
Until technical training, university, and post-	1.582 (0.688-1.948)	0.097
**Age**		
20-47 years (1st quartile)	1	-
48-57 years (2nd quartile)	1.234 (0.666-2.286)	0.504
58-65 years (3rd quartile)	0.933 (0.480-1.812)	0.733
65 years or more (4th quartile)	1.138 (0.542-2.390)	0.733
**MMSE**		
Normal	1	-
Altered	0.335 (0.128-0.873)	0.025
**MOCA**		
Normal	1	-
Altered	0.858 (0.209-3.517)	0.831
**Clinical evaluation of swallowing**		
Normal	1	-
Altered	1.772 (1.094-2.872)	0.020

**Caption:** MMSE – Mini-Mental State Examination; MoCA – Montreal Cognitive Assessment; PR – prevalence ratio; CI 95% - confidence interval adopted; p-valor – level of significance adopted.

## DISCUSSION

Factors associated with frailty in patients with neurodegenerative diseases were identified through multivariate regression in this study. We found that the prevalence of frailty was greater amongst those who had oropharyngeal dysphagia and less present among individuals with cognitive alterations.

The greater prevalence of frail individuals with neurodegenerative diseases and oropharyngeal dysphagia shows how important it is for healthcare professionals to focus on digestive and dietary questions, which seem to be the first factors associated with frailty in this specific population^(23,24)^. Dysphagia presents diverse associated consequences, that can result in dehydration, malnutrition, increased hospitalization, and early mortality^(8,10)^. Due to the severity and possible repercussions of these cases, coordinated care must be established and represent a priority in healthcare^(24)^.

The association between frailty and oropharyngeal dysphagia is described in the literature, however, the causal relationship between them is not established. At the same time that oropharyngeal dysphagia is associated with malnutrition, aspiration pneumonia, and early mortality, frailty is associated with functional decline, sarcopenia amongst other comorbidities, and consequences such as difficulty in swallowing. It is understood that the outcome of oropharyngeal dysphagia can be associated with exposure to frailty, while the frailty outcome can be associated with exposure to dysphagia^(5,25)^. Reverse causality should be considered and suggests that longitudinal studies and those that investigate the natural history of the disease be developed. An important result is already identified in this study, given that the prevalence of frailty is greater amongst those who have dysphagia. This demonstrates that they can be simultaneously related and result in significant harm to this population and should represent a healthcare priority.

Studies show that frailty measures, including grip strength and walking speed were associated with an increase in morbidity and mortality of patients with oropharyngeal dysphagia^(5)^, which corroborates with the phenotypic components of frailty, including unintentional weight loss, self-reporting of fatigue and/or exhaustion, reduction in muscular force, slow walking speed and low level of physical activity^(8-10)^. This shows once again, how they are related to one another and that both should be evaluated early on by healthcare professionals. In identifying an individual with a neurodegenerative diagnosis, the importance of a multidisciplinary team, as well as the assessment of swallowing and frailty is highlighted^(7)^.

Studies relating tongue pressure, handgrip force, arm and calf circumference, and walking speed are being associated with frailty^(26-28)^. This shows that already structured protocols, used to measure the degree of frailty of individuals in this study, are important to give healthcare professionals a general idea of how fragile the individual is. However, these protocols do not cover the evaluation of all the suggestive signs of sarcopenia and, consequently, frailty^(29)^. Additionally, instruments that involve signs and symptoms for tracking and future diagnosis of oropharyngeal dysphagia are pertinent considering the resulting aggravating factors for health.

It is possible to observe that in this study 41.1% of the individuals analyzed, have a neurodegenerative disease of genetic origin, more precisely Spinocerebellar ataxias. This finding, however, does not agree with the literature, which highlights a greater prevalence for the movement disorder subgroup in this population, with neurogenetic disorders being the least prevalent^(30)^. Due to the convenience sample (a limiting factor in this study), this data could be a little unusual, and given this, we highlight the importance of further research with such individuals to understand all the possible scenarios and factors associated with frailty in this population.

The use of the Edmonton Scale is another limitation to be highlighted. Despite being a constantly used and validated scale for the geriatric population, it is not validated in Brazil for patients with neurodegenerative disease. Regardless, the scale stands out for its range in monitoring physical and psychological symptoms, being a valid and widely used instrument for allowing customized care for each patient based on the scores presented, highlighting the need for new interventions^(18)^.

Additionally, the prevalence of frailty in this population is lower in the participants who presented cognitive alterations. These results need to be analyzed with greater care, larger sample sizes, and robust statistics to understand this association. A recent study found that frailty and cognitive impairment were independently associated with recurrent falls in non-institutionalized elderly individuals. There was a lack of synergistic effect between frailty and cognitive impairment^(31)^. Prior studies have shown that frailty is associated with adverse health outcomes and low cognitive function^(32)^, however, they demonstrated that pre-frailty is not associated with cognitive impairment, only with its more advanced phases^(33)^, which suggestively corroborates with the findings of this study, given that there is a lower prevalence of frailty in the cognitive impairments described by the MMSE. In addition, the study indicated that the cognitive state offered additional discriminatory power in differentiating the elderly in advanced stages of frailty at risk of incapacity in daily activities and mortality^(34)^.

Regarding the difference observed between the MMSE and MoCA results, the difference in diagnostic accuracy of each instrument was suggested. MMSE is the most commonly used screening test in clinical practice, but it lacks sensitivity when identifying light cognitive impairment and non-amnestic cognitive impairment compared with the MoCA. Given that it evaluates executive functions, the MoCA is recommended for screening for impairment in individuals with cerebrovascular deficiencies, chronic kidney disease, and diabetes mellitus. In all these pathologies in which cognitive impairment involved subcortical structures of the Nervous System, the performance of MoCA was found to be superior to that of the MMSE compared with the cognitive evaluation with broad neuropsychological testing^(35,36)^.

Finally, it is important to identify individuals with frailty early on, since this allows for planning of healthcare interventions, including early speech-language therapy care, reducing the chances of irreversible complications for frail individuals.

## CONCLUSION

In this study, we found that there was a greater prevalence of frailty in neurodegenerative individuals with oropharyngeal dysphagia, while adequate cognitive performance presented a lower prevalence of frailty, which was another protective factor. Specific measures and qualifications of care should be implemented in healthcare services, seeking early diagnosis and timely rehabilitation.
