# Supplementary material for: Factors associated with frailty in patients with neurodegenerative diseases
Source: Codas. 2022 Jun 17;34(5):e20200214. doi: 10.1590/2317-1782/20212020214 (PMC9886181; doi:10.1590/2317-1782/20212020214)
Supplement: Translation

# Translation: Fatores associados a fragilidade em pacientes com doenças neurodegenerativas

**Authors:** Rafaela Soares Rech, Marina Martins Pereira Padovani, Nathalia Flores Oliveira, Bruna Graciele Souza Alós, Annelise Ayres, Maira Rozenfeld Olchik

**Affiliations:** 1 Universidade Federal de Ciências da Saúde de Porto Alegre - Porto Alegre (RS), Brasil.; 2 Faculdade de Ciências Médicas Santa Casa de São Paulo - São Paulo (SP), Brasil.; 3 Universidade Federal do Rio Grande do Sul – UFRGS - Porto Alegre (RS), Brasil.; 4 Hospital de Clínicas de Porto Alegre - Porto Alegre (RS), Brasil

**Keywords:** Fragilidade, Envelhecimento, Transtornos da Deglutição, Cognição, Fonoaudiologia

## Abstract

**Objetivo:**

Identificar os fatores associados à fragilidade em pacientes com doenças neurodegenerativas.

**Método:**

Estudo transversal, cuja amostra foi composta por 150 pacientes com diagnóstico de doenças neurodegenerativas atendidos em um ambulatório de Fonoaudiologia de um hospital de referência no sul do Brasil. Foi realizada análise secundária exploratória dos prontuários dos pacientes atendidos neste ambulatório entre o período de abril de 2016 e maio de 2019. As informações coletadas foram: sexo, idade, escolaridade, tipo de doença neurodegenerativa, tempo de doença, fragilidade (Edmonton Frail Scale - EFS), deglutição (Northwestern Dysphagia Patient CheckSheet- NDPCS, Eating Assessment Tool-EAT 10) e cognição (Mini-Mental State Examination-MMSE e Montreal Cognitive Assessment-MoCA). As variáveis quantitativas contínuas foram analisadas a partir de média e desvio padrão e as quantitativas categóricas a partir de frequência absoluta e relativa, assim como analisou-se a associação destas com o desfecho pelo teste Qui-Quadrado. As Razões de Prevalência brutas e ajustadas foram avaliadas a partir da Regressão de Poisson com variância robusta. Todos os testes estatísticos foram considerados significativos a um nível de 5%.

**Resultados:**

Os fatores significativos associados à fragilidade foram à presença de disfagia orofaríngea e desempenho cognitivo alterado. Indivíduos com a fragilidade apresentam maior prevalência de disfagia orofaríngea (RP=1,772(1,094-2,872)), enquanto a cognição alterada está associada a menor prevalência de fragilidade (RP=0,335(0,128-0,873).

**Conclusão:**

A disfagia orofaríngea pode ser um importante fator clínico preditivo a ser considerado em casos de fragilidade em pacientes com doenças neurodegenerativas.

## INTRODUÇÃO

As doenças neurodegenerativas, entre elas: Doença de Alzheimer, Doença de Parkinson, Doenças de Huntington, Esclerose Lateral Amiotrófica, Demência Frontotemporal e Ataxias Espinocerebelares^(1)^, repercutem na diminuição da funcionalidade de diversos sistemas do corpo, de acordo com as características específicas de cada patologia. Estas apresentam características semelhantes: função mitocondrial prejudicada com aumento de danos oxidativos, defeitos no sistema ubiquitina-proteassoma, presença anormal de proteínas agregadas e alterações no metabolismo do ferro^(2,3)^. Esses fatores são parte de um ciclo repetitivo, o qual a ação de qualquer um deles, pode iniciar a lesão do neurônio, que por sua vez, “recruta” os outros fatores, com o propósito de destruir a célula^(4)^.

A neurodegeneração é uma característica debilitante e incurável, que tem sido evidenciada na literatura com um aumento exponencial de sua prevalência^(1)^. As principais consequências fisiológicas relacionadas a este estado neuronal repercutem nas funções das atividades de vida diária e nas funções vitais do ser humano, principalmente nas tarefas que exigem complexidade motora, cognitiva, de deglutição e de fala^(3,5)^. Pessoas de todas as idades podem ser diagnosticadas com doenças neurodegenerativas, entretanto, está é muito mais prevalente em idosos^(6)^.

Entre os diversos agravos decorrentes das consequências relacionadas aos prejuízos neuronais produzidos e aos declínios das habilidades humanas na população específica está a fragilidade^(7)^. A fragilidade tem caráter multisistêmico e é compreendida por um fenótipo que inclui cinco componentes principais: perda de peso não intencional, autorrelato de fadiga e/ou exaustão, diminuição de força muscular, lentidão na velocidade de marcha e baixo nível de atividade física^(8,9)^. Atualmente, a fragilidade é vista como um tipo de síndrome que possui uma base específica e independente, podendo ser evitada quando identificada precocemente ou, quando intervinda nas bases causadoras, ao menos postergada^(10,11)^.

Atualmente, estudos buscam identificar quais são os aspectos que resultam no curso mais negativo de uma doença neurodegenerativa. Alguns autores indicam que a incidência de fragilidade é mais comum em pacientes com Doença de Parkinson do que na comunidade em geral e estão associadas a um curso mais adverso da doença^(12)^. O mesmo ocorre com a Doença de Alzheimer e com idosos independentes da comunidade que são populações em risco, visando a minimização da fragilidade na expectativa de modificar o impacto nas doenças degenerativas e suas implicações^(5,13)^.

Diante a lacuna existente na literatura indexada sobre as características mais prevalentes na fragilidade e seus fatores associados em indivíduos com doenças neurodegenerativas, bem como as consequências e peculiaridades advindas do quadro clínico específico desta população, seus preditores e achados clínicos são potencializadores de cuidados coordenados, reabilitação específica e interprofissional^(14)^. Além disso, acredita-se que através de estratégias oportunas, é possível proporcionar melhora na saúde global e na qualidade de vida destes indivíduos, bem como diminuição dos custos em saúde^(15)^.

Este estudo tem como hipótese que a disfagia orofaríngea é o principal fator associado à fragilidade em pacientes com doenças neurodegenerativas visto as características funcionais e agravos existentes nestes casos na prática clínica ambulatorial. Sendo assim, objetiva-se identificar os fatores associados à fragilidade em pacientes com doenças neurodegenerativas.

## MÉTODO

Trata-se de um estudo transversal com uma amostra de conveniência, intencional consecutiva, com dados secundários dos prontuários de pacientes com doenças neurodegenerativas atendidos em um ambulatório de Fonoaudiologia de um hospital de referência na cidade de Porto Alegre - RS entre abril de 2016 e maio de 2019. Foram excluídos os dados dos pacientes que não apresentavam avaliação fonoaudiológica completa ou que os dados não estivessem completos no prontuário.

Na perspectiva de estimar o número necessário de prontuários a serem analisados, planejou-se o cálculo do tamanho amostral baseado em um estudo que associou fragilidade e cognição^(16)^. Definiu-se um poder de estudo de 90% e nível de significância de 0,05. Para compensar possíveis perdas, aumentou-se a amostra em 10%. O tamanho da amostra necessário foi definido, portanto, em 150 indivíduos.

As variáveis contextuais utilizadas nesse estudo foram: sexo (feminino; masculino) idade (20-47 anos; 48-57 anos; 58-65 anos; 65 anos ou mais), escolaridade (até ensino fundamental; até ensino médio incompleto; até ensino médio; até ensino técnico, ensino superior), cognição (normal; alterada) e deglutição (normal; alterada). As categorias foram estabelecidas a partir da distribuição da amostra.

A fragilidade foi identificada a partir da Escala de Fragilidade Edmonton^(17)^ (EFS), traduzida e validada para o português-brasileiro. Apesar de não ser utilizada frequentemente por casos neurodegenerativos e sim casos geriátricos, essa escala tem sido amplamente utilizada mundialmente e é considerada uma escala muito abrangente em seus domínios de investigação, completa, com boa acurácia diagnóstica, de fácil aplicação e aplicável por diversos profissionais^(18)^. Visto a magnitude da escala e a ausência para a população específica, esta escala foi utilizada e os participantes foram diagnosticados por fonoaudiólogas especialistas na área e que trabalham no setor ambulatorial deste hospital. Trata-se de uma escala de avaliação de fragilidade em idosos e que compreende nove domínios: cognição, estado geral de saúde, independência funcional, uso de medicamentos, nutrição, humor, continência urinária e desempenho funcional, investigados em 11 itens. A pontuação varia de 0 a 17 pontos, sendo essa a pior fragilidade. Os escores para a análise são: 0-4 pontos, não apresenta fragilidade; 5-6 pontos, aparentemente vulnerável; 7-8 pontos, fragilidade leve; 9-10 pontos, fragilidade moderada; 11 ou mais, fragilidade severa.

A avaliação da deglutição foi composta por avaliação clínica a partir do instrumento *Northwestern Dysphagia Patient Check Sheet* (NDPCS), traduzido e adaptado para o português-brasileiro^(19)^. Caracteriza-se por uma avaliação clínica breve e funcional da deglutição composta por 28 itens divididos em cinco categorias: história médica, aspectos comportamentais, função motora ampla, teste motor oral e observação durante as provas de deglutição. As provas de deglutição foram realizadas a partir da oferta de livre demanda da consistência líquida, onde foram observados sinais sugestivos de penetração/aspiração laringotraqueal segundo as orientações do protocolo utilizado e as recomendações internacionais para a prática clínica fonoaudiológica. O diagnóstico fonoaudiológico foi considerado como normal quando não havia riscos e alterado quando possibilidade de penetração e/ou aspiração laringotraqueal. A categorização se deu de acordo com a distribuição da amostra.

A avaliação de autopercepção de disfagia orofaríngea foi realizada a partir do *Eating Assessment Tool* (EAT-10)^(20)^. Trata-se de um instrumento de autopercepção da identificação do risco para disfagia. Tem como objetivo a indicação e intervenção multiprofissional o mais precoce possível. É composto por dez questões simples, que fornecem informações sobre funcionalidade, impacto emocional e sintomas físicos que a disfagia pode trazer ao indivíduo. Com pontuação 3 ou mais considera-se que o participante está sob risco de disfagia orofaríngea.

A avaliação cognitiva foi realizada a partir do Mini Exame do Estado Mental (MEEM)^(21)^ e *Montreal Cognitive Assessment* (MoCA)^(22)^, ambos validados e traduzidos para o português-brasileiro. Trata-se de testes de rastreio cognitivo que visam verificar rapidamente as funções cognitivas. O MEEM avalia, em suas 11 tarefas, funções de: orientação temporo-espacial, memória, atenção, cálculo, linguagem e praxia construtiva. Os 11 itens são divididos em duas seções, onde uma exige respostas verbais a questões de orientação, memória e atenção, e a outra avalia leitura e escrita, englobando habilidades de nomeação, comandos verbais e escritos e capacidade de copiar um desenho. A pontuação varia de 0 a 30 pontos. São considerados valores medianos dentro do padrão de normalidade pontuação igual ou superior a 29 pontos para indivíduos com escolaridade superior a 11 anos; 28 pontos para indivíduos de 9 a 11 anos de escolaridade; 26,5 pontos para indivíduos de 5 a 8 anos de escolaridade; 25 pontos de 1 a 4 anos de escolaridade e 20 pontos para analfabetos.

O MoCA, por sua vez, avalia oito funções, dentre elas: apraxia visuoespacial, nomeação, memória, atenção, linguagem, abstração e orientação. Possui pontuação total de 30 pontos, sendo considerada função cognitiva normal os indivíduos que apresentarem pontuação igual ou superior a 24 pontos. Tanto o MEEM quanto o MoCA foram considerados normais ou alterados de acordo com as respostas esperadas para cada indivíduo de acordo com a sua escolaridade (se superior ou inferior, respectivamente).

### Análise estatística

A análise descritiva dos dados foi realizada de acordo com as distribuições de cada variável. Para a análise das variáveis categóricas foram utilizadas as frequências absolutas e relativas e para a análise das variáveis quantitativas, utilizou-se média e desvio padrão devido à normalidade dos dados, analisada pelo teste de Kolmogorov-Smirnov e pela análise visual de histogramas para complementação. A correlação de Spearman foi realizada para testar a correlação das variáveis e verificar ausência do viés de aferição. Visto que todas as variáveis apresentaram valor de correlação muito fraca (0 a 0,3), fraca (0,3 a 0,5) e moderada (0,5 a 0,7), foram acrescentadas no modelo teórico deste estudo. Além disso, avaliou-se a presença de multicolinearidade por meio das estimativas do fator de inflação de variância (VIF), observando que os pontos de corte são bons (próximos a 1) indicando que as variáveis não são multicolineares. O teste Qui-quadrado foi utilizado para avaliar as associações nas variáveis estudadas com nível de significância de p ≤0,05. Para calcular as relações entre as variáveis, levando em consideração possíveis fatores de confusão utilizou-se o teste Regressão de Poisson com variância robusta, a partir da Razão de Prevalência (RP) e seus respectivos intervalos de confiança de 95%. No modelo ajustado, incluiu-se variáveis teoricamente relevantes. Sendo que, foram incluídas todas as variáveis no modelo multivariável final, visto que esse estudo tem caráter exploratório para identificar como as variáveis se comportam em casos neurodegenerativos. Os resultados foram analisados estatisticamente pela versão 18.0 do programa *Statistical Package for the Social Sciences* (SPSS).

### Aspectos éticos

Este estudo foi aprovado pelo Comitê de Ética em Pesquisa (CEP) da instituição sob número 1803-23. Todos os indivíduos envolvidos na pesquisa assinaram o Termo de Consentimento Livre e Esclarecido.

## RESULTADOS

Ao total, 150 prontuários foram incluídos no estudo. Destes 62 (41%) dos indivíduos possuíam doença neurodegenerativa de origem genética, mais precisamente as ataxias espinocerebelares, seguido de doenças neuromusculares 50 (33,7%), doenças do distúrbio do movimento 21 (14%) e 17 (11,3%) casos de acidente vascular encefálico.

Na tabela 1 encontra-se a caracterização da amostra e a sua associação bruta (bivariada). A maioria dos indivíduos era do sexo feminino 78 (52,0%), além disso, as mulheres apresentavam maior prevalência de fragilidade 41 (52,6%). A média de idade foi de 54,13 (± 8,41) anos, a escolaridade, medida em anos foi de 8,74 (± 1,21) anos e tempo médio de doença foi de 9,75 (± 2,34) anos. A prevalência de alteração da deglutição diagnosticada pela avaliação clínica foi de 42,0%, enquanto as alterações cognitivas foram de 22,6% da amostra estudada. Mantiveram-se associadas à fragilidade as variáveis: sexo (p<0,000), alteração de deglutição (p<0,001) e alterações cognitivas (p<0,001).

**Tabela 1:** Caracterização da amostra e pontuação nas avaliações de fragilidade, deglutição e cognição. Porto Alegre, 2018 (n=150)

**Variáveis**	**Sem fragilidade**	**Com Fragilidade**	**p-valor**
**Sexo**			<0,001
Feminino	37 (47,4%)	41 (52,6%)	
Masculino	52 (72,2%)	20 (27,8%)	
**Escolaridade**			0,227
Até ensino fundamental	25 (56,8%)	19 (43,2%)	
Até ensino médio incompleto	18 (52,9%)	16 (47,1%)	
Até ensino médio	24 (72,7%)	9 (27,3%)	
Até ensino técnico, superior ou pós	21 (55,3%)	17 (44,7%)	
**Idade**			0,243
20-47 anos (1º quartil)	27 (67,5%)	13 (32,5%)	
48-57anos (2º quartil)	17 (47,2%)	19 (52,8%)	
58-65 anos (3º quartil)	21 (60,0%)	14 (40,0%)	
65 anos ou mais (4º quartil)	22 (56,4%)	17 (43,6%)	
**MEEM**			<0,001
Normal	51 (51,5%)	48 (48,5%)	
Alterado	38 (74,5%)	13 (25,5%)	
**MOCA**			0,019
Normal	66 (56,9%)	50 (43,1%)	
Alterado	23 (67,6%)	11 (32,4%)	
**Avaliação clínica da deglutição**			<0,001
Normal	60 (68,9%)	27 (31,1%)	
Alterado	21(33,3%)	42 (66,7%)	

**Legenda:** n – número da amostra; MEEM – Mini Exame do Estado Mental; MoCA – *Montreal Cognitive Assessment*.

Na tabela 2 encontram-se as razões de prevalências multivariáveis estimadas a partir da análise exploratória por controle de possíveis fatores de confusão. Mantiveram-se significativamente associadas à fragilidade a disfagia orofaríngea e a alteração cognitiva, sendo que a prevalência de fragilidade é maior entre os que têm disfagia (RP=1,772(1,094-2,872)) e menor nos que têm alterações cognitivas (RP=0,335(0,128-0,873)).

**Tabela 2:** Razões de prevalência ajustadas em pacientes com fragilidade e doenças neurodegenerativas. Porto Alegre, 2018 (n=150)

**Variáveis**	**Fragilidade** **RP (IC)**	**p-valor**
**Sexo**		
Feminino	1	-
Masculino	0,606 (0,339-1,083)	0,091
**Escolaridade**		
Até ensino fundamental	1	-
Até ensino médio incompleto	1,158(0,688-1,948)	0,581
Até ensino médio	0,706(0,307-1,624)	0,413
Até ensino técnico, superior ou pós	1,582(0,688-1,948)	0,097
**Idade**		
20-47 anos (1º quartil)	1	-
48-57anos (2º quartil)	1,234(0,666-2,286)	0,504
58-65 anos (3º quartil)	0,933(0,480-1,812)	0,733
65 anos ou mais (4º quartil)	1,138(0,542-2,390)	0,733
**MEEM**		
Normal	1	-
Alterado	0,335(0,128-0,873)	0,025
**MOCA**		
Normal	1	-
Alterado	0,858(0,209-3,517)	0,831
**Avaliação clínica da deglutição**		
Normal	1	-
Alterado	1,772(1,094-2,872)	0,020

**Legenda:** MEEM – Mini-Exame do Estado Mental; MoCA – *Montreal Cognitive Assessment*; RP – razão de prevalência; IC 95% - intervalo de confiança adotado; p-valor – nível de significância adotado.

## DISCUSSÃO

Fatores associados à fragilidade em pacientes com doenças neurodegenerativas, através de regressão multivaríavel, foram identificados neste estudo. Revelou-se que a prevalência de fragilidade é maior entre os que têm disfagia e menor nos que têm alterações cognitivas.

A maior prevalência de indivíduos frágeis com doenças neurodegenerativas e disfagia orofaríngea revela o quanto é importante que profissionais da saúde se preocupem com as questões alimentares e digestivas, que parecem ser, os primeiros fatores associados a fragilidade nesta população específica^(23,24)^. A disfagia por sua vez, apresenta diversas consequências associadas, que podem resultar em desidratação, desnutrição, aumento de hospitalização e mortalidade precoce^(8,10)^. Devido a gravidade e as possíveis repercussões destes casos é necessário que cuidados coordenados sejam bem estabelecidos e representem prioridade nos cuidados em saúde^(24)^.

A associação entre fragilidade e disfagia orofaríngea está descrita na literatura, entretanto, não está estabelecida a relação causal entre estas. Ao mesmo tempo em que a disfagia orofaríngea está associada à desnutrição, pneumonia aspirativa e mortalidade precoce, a fragilidade está associada ao declínio funcional, sarcopenia entre outras comorbidades e consequências como a dificuldade na deglutição. Sabe-se que o desfecho disfagia orofaríngea pode estar associada à exposição fragilidade, quanto o desfecho fragilidade estar associado à exposição disfagia^(5,25)^. A causalidade reversa deve ser considerada e sugere que estudos longitudinais e que investiguem a história natural da doença sejam desenvolvidos. De toda forma, importante resultado é identificado neste estudo, sendo que a prevalência de fragilidade é maior entre os que têm disfagia, demonstrando que estes podem estar concomitante relacionados e resultarem em importantes prejuízos a esta população e deve representar prioridade nos cuidados em saúde.

Estudos evidenciam que medidas de fragilidade, incluindo força de preensão palmar e velocidade de caminhada foram associadas ao aumento da morbidade e mortalidade de pacientes com disfagia orofaríngea^(5)^, o que corrobora com os componentes do fenótipo da fragilidade, sendo eles, perda de peso não intencional, autorrelato de fadiga e/ou exaustão, diminuição de força muscular, lentidão na velocidade de marcha e baixo nível de atividade física^(8-10)^. Demonstrando mais uma vez, como estas estão relacionadas entre si e que ambas devem ser avaliadas precocemente por profissionais da saúde. Ao identificar um indivíduo com diagnóstico neurodegenerativo salienta-se a importância de uma equipe multiprofissional, assim como da avaliação da deglutição e da fragilidade^(7)^.

Estudos relacionando a pressão de língua, pressão palmar, circunferência de braço e panturrilha, assim como velocidade de caminhada estão sendo associados à fragilidade^(26-28)^ demonstrando que protocolos já estruturados, como o utilizado para dimensionar o grau de fragilidade dos indivíduos neste estudo, são importantes para dar ao profissional da saúde uma noção geral do quão frágil o indivíduo é. Contudo, estes protocolos não contemplam a avaliação de todos os sinais sugestivos de sarcopenia e, consequentemente, fragilidade^(29)^. Além disso, instrumentos que envolvam sinais e sintomas para o rastreamento e futuro diagnóstico de disfagia orofaríngea são pertinentes considerando os agravos em saúde resultantes.

É possível observar que neste estudo 41,4% dos indivíduos analisados, possuíam doença neurodegenerativa de origem genética, mais precisamente as ataxias espinocerebelares. Este dado, no entanto, não vai ao encontra da literatura que aponta uma maior prevalência do subgrupo dos distúrbios do movimento nesta população, sendo as neurogenéticas as menos prevalentes^(30)^. Devido a amostra de conveniência (fator limitante deste estudo), esse dado pode ser um pouco peculiar, por isso ressaltamos a importância de mais estudos como estes para entender todos os possíveis cenários e fatores associados a fragilidade nesta população.

Outra limitação a ser destacada é o uso da Escala de Edmonton. Apesar de ser uma escala constantemente utilizada e validada para a população geriátrica, está não é validada no Brasil para pacientes com doença neurodegenerativa. De toda forma, a escala destaca-se por sua abrangência em monitorar sintomas físicos e psicológicos, sendo um instrumento válido e amplamente utilizado para possibilitar cuidados diferenciados a cada paciente a partir dos escores apresentados, documentando a necessidade de intervenções, avaliando sua eficácia ou identificando a necessidade de novas intervenções^(18)^.

Além disso, nessa população a prevalência de fragilidade é menor nos participantes que tem alterações cognitivas. Resultados que precisam ser analisados com maior cautela, amostras maiores e estatísticas robustas para compreender essa associação. Um estudo recente identificou que a fragilidade e o comprometimento cognitivo foram independentemente associados a quedas recorrentes em idosos não institucionalizados. Houve uma falta de efeito sinérgico entre fragilidade e comprometimento cognitivo^(31)^. De toda forma, estudos prévios demonstraram que a fragilidade está associada a resultados adversos à saúde e baixa função cognitiva^(32)^, porém demonstram que a pré-fragilidade não está associada ao comprometimento cognitivo, apenas em suas fases mais avançadas^(33)^, o que corrobora sugestivamente com os achados deste estudo, visto que há menor prevalência de fragilidade nos comprometimentos cognitivos descritos pelo MEEM. Ademais, um estudo indicou que o estado cognitivo ofereceu poder discriminatório adicional na diferenciação de idosos em estágios avançados de fragilidade em risco incapacidade nas atividades da vida diária e mortalidade^(34)^.

Quanto a diferença encontrada entre os resultados do MEEM e do MoCA sugere-se a diferença na acurácia diagnóstica de cada instrumento. O MEEM é o teste de rastreio mais utilizado na prática clínica, mas é pouco sensível para identificar o comprometimento cognitivo leve e o comprometimento cognitivo não amnéstico comparado ao MoCA. O MoCA por avaliar funções executivas, vem sendo indicado para o rastreio de comprometimento em indivíduos com insuficiência cerebrovascular, doença renal crônica e diabetes melitus. Em todas essas patologias nas quais o comprometimento cognitivo envolve estruturas subcorticais do Sistema Nervoso, o desempenho do MoCA mostra se superior ao do MEEM comparados à avaliação cognitiva com baterias neuropsicológicas amplas^(35,36)^.

Por fim, identificar precocemente indivíduos com fragilidade é importante, pois permite o planejamento de ações de saúde, incluindo o atendimento fonoaudiológico precoce, reduzindo as chances de complicações irreversíveis para sujeitos frágeis.

## CONCLUSÃO

Concluiu-se neste estudo que houve maior prevalência fragilidade em indivíduos neurodegenerativos com disfagia orofaríngea, sendo que o desempenho cognitivo satisfatório apresenta menor prevalência de fragilidade, sendo um fator protetivo. Medidas específicas e qualificação do cuidado devem ser implementadas nos serviços de saúde visando diagnósticos precoces e reabilitações oportunas.
